# Army and Navy Dentists

**Published:** 1860-01

**Authors:** 


ARMY AND NAVY DENTISTS.—There seems to be some prospect that the army and navy of the United States will soon be provided with educated dentists, the need of which has long been felt by both services. Dr. Maynard has, for some time past, urged the great importance of the subject upon the government; and it is said to have met with the favorable consideration both of the surgeon-general of the army and of the secretary of the navy.

